# Selective lung ventilation in pediatric pulmonary hydatid cyst surgery: a comparative analysis of operative efficiency and clinical outcomes

**DOI:** 10.1007/s00383-026-06346-1

**Published:** 2026-02-23

**Authors:** Süleyman Arif Bostancı, Elif Emel Erten, İrem Akbaş, Selman Kürşat Balcı, Sabri Demir, Ahmet Ertürk, Can İhsan Öztorun, Sengül Özmert, Emrah Şenel, Müjdem Nur Azılı

**Affiliations:** 1 Ankara Yıldırım Beyazıt University, Faculty of Medicine, Department of Pediatric Surgery, Ankara Bilkent City Hospital, Childrens’ Hospital, Department of Pediatric Surgery, Universiteler Boulvard 1604. Street, Çankaya, Ankara 06800 Turkey; 2 Ankara Bilkent City Hospital, Childrens’ Hospital , Department of Pediatric Surgery, Ankara, Turkey; 3 Ankara Bilkent City Hospital, Childrens’ Hospital, Department of Pediatric Surgery, Health Science University, Ankara, Turkey; 4 Ankara Yıldırım Beyazıt University, Faculty of Medicine, Department of Pediatric Surgery, Ankara, Turkey; 5 Ankara Bilkent City Hospital, Childrens’ Hospital, Department of Pediatric Anaesthesiology, Health Science University, Ankara, Turkey

**Keywords:** Selective ventilation, Hydatid cyst, Children, Lung

## Abstract

**Purpose:**

Selective lung ventilation (SLV) during thoracic surgery improves surgical exposure and prevents contamination of the contralateral lung. In pediatric pulmonary hydatid cyst surgery, bronchopulmonary fistulas complicate surgical visualization and repair, significantly increasing the risk of contamination. This study aimed to evaluate the effectiveness of SLV in terms of operative time, prolonged air leak, reoperation necessity, and hospital stay duration in pediatric thoracotomy cases performed for hydatid lung cysts.

**Methods:**

This retrospective study included pediatric patients under 18 years of age who underwent thoracotomy for pulmonary hydatid cysts between 2014 and 2022. SLV was performed with double-lumen tubes in children aged seven years and older and with endobronchial blockers in younger children. Patients were grouped according to the type of ventilation (SLV or non-selective lung ventilation, NSLV), and variables such as cyst volume, operative time, postoperative complications, and hospital stay duration were compared.

**Results:**

A total of 86 patients were analyzed, with 60 in the NSLV group (69.8%) and 26 in the SLV group (30.2%). Despite significantly larger cyst volumes in the SLV group (221.6 cm³ vs. 104.9 cm³, *p* = 0.048), SLV patients had notably shorter operative times (115.7 vs. 153.7 min, *p* = 0.001) and significantly reduced hospital stays (7.2 vs. 12.3 days, *p* = 0.05). No SLV-related complications such as atelectasis or infection were observed.

**Conclusion:**

This first pediatric-focused study demonstrates that SLV effectively reduces operative time and hospitalization without increasing complication rates, suggesting it as a reliable option in pediatric hydatid cyst surgery.

## Introduction

Intraoperative one-lung isolation is a surgical technique that has been utilized since the 1980 s to facilitate surgical exposure during intrathoracic surgeries, particularly those that focus on the lungs and airway malformations [1–3]. Additionally, this approach is advantageous for preventing the contralateral lung from soiling. The use of double-lumen endobronchial tubes (EBTs) due to technological advancements has made possible selective intubation. Double-lumen tubes are a viable option for selective bronchial intubation in children over the age of 6–8 years for one-lung ventilation [3]. However, this technology has limited use among younger children. In recent years, many varieties of endobronchial blockers have made one-lung ventilation a viable option for young children [4–8].

Pulmonary hydatid cyst is a zoonotic disease caused by Echinococcus species. This disease remains a significant public health concern, particularly in regions where it is endemic [9]. While lung involvement is second only to liver involvement in hydatid disease, there has been an increasing prevalence in children [10–12]. In most cases, pulmonary hydatid cysts are associated with Echinococcus granulosus. Typically, diagnosis is made based on symptoms resulting from cyst expansion or allergic reactions. There is no standard treatment protocol for pulmonary hydatid cysts due to their variable localization and effects. The most common treatment modality is surgical intervention via thoracotomy or thoracoscopic approach [13, 14]. The surgical procedure involves the excision of the germinal membranes within the cyst and the repair of any cystobronchial fistula that may have developed. The porous tissue structure of the lung allows cysts to expand beyond the capacity of the liver. However, visualizing and repairing cystobronchial fistulas can be challenging due to air leakage during surgery. Furthermore, the presence of multiple high-flow bronchopulmonary fistulas can result in significant oxygenation issues due to respiratory decompensation [15].

Pulmonary cystic echinococcosis poses a significant public health concern in endemic regions and is frequently encountered in pediatric surgical practice. The objective of our study is to conduct a comparative analysis of cases that underwent selective ventilation and those that did not, with a focus on variables such as operative time, the incidence of postoperative persistent bronchopleural fistula, the necessity for additional interventions, and the duration of hospital stay.

## Materials and methods

This study was designed as a retrospective, single-center investigation. The medical records of pediatric hydatid cysts of the lung cases treated by thoracotomy at a tertiary care institution between January 2014 and December 2022 were reviewed following Ethics Committee approval number E2-23–3831. The surgical and anesthesia teams collaborated to determine the need for selective lung ventilation. Double-lumen tubes were utilized for selective lung ventilation in children aged 6–8 years and older. For younger children under 6 years old, when double-lumen tubes were not feasible, bronchial blockade was achieved with a Coopdech Endobronchial Bloker Tube (Smith Medical, Rosmolen, NL) advanced through the endotracheal tube under endoscopic guidance. The fiberoptic bronchoscope’s outer diameter of 2.8 mm enabled the administration of blockers with a minimum diameter of 3.0 mm and larger. Patients with difficult airways and those who required tracheal intubation less than 3.0 mm owing to the outer diameter of the fiberoptic bronchoscope were excluded from the study. Patients were divided into two subgroups: those who received selective pulmonary ventilation and those who did not.

Electrocardiography, peripheral blood oxygen saturation, and non-invasive blood pressure were performed in the operating room to monitor all patients. The operation was completed with a standard intubation tube in patients who did not require selective lung ventilation after induction of anesthesia. A double-lumen tube was inserted under auscultatory control or by video laryngoscopy in patients aged 6–8 years who required selective lung ventilation. The location and duration of the bronchial blocker were determined according to the surgical requirements.

Surgical treatment was performed via thoracotomy and consisted of cyst drainage, removal of the germinal membrane, and subsequent repair of air leaks. In the selective ventilation group, the bronchial occlusion was opened in a controlled manner after surgical repair to check that the air leak and atelectasis in the lung had been eliminated.

Mean, standard deviation, and median (range) were used for quantitative variables; number of patients and percentages were used for qualitative variables. The Kolmogorov-Smirnov test was used to evaluate the distribution of numerical variables. The Mann-Whitney U test or Student’s t-test was used to compare two independent groups. The chi-squared test was used to assess differences between categorical variables. All analyses were performed with the Statistical Package for the Social Sciences (SPSS) for Windows version 25.0. A p-value of less than 0.05 was accepted as the level of statistical significance.

## Results

Between 2014 and 2022, 86 patients who underwent surgery for pulmonary hydatid lesions were included in this retrospective study. The mean age of the patients was 12 years, with a range of 2–17 years. Thirty-one patients were female (36%). Of these patients, 50 patients were treated for hydatid cysts in the right lung and 36 for those in the left lung. The mean volume of the hydatid cysts was 140.19 cm³ (range: 2.72–2190.2.72.2). Suspected cyst rupture was a significant preoperative observation in 20 (23.2%) patients. This could increase the urgency and complexity of the surgical procedure. Four patients had persistent air leaks (PAL) for more than one week, and three required reoperations for stage-4 PAL (3.4%), indicating the complexity of surgical treatment. The mean operative time was 142.2 min (range 60–210), and the mean length of hospital stay was 10.8 days (range 4–40).

Of all patients, 26 patients (30.2%) underwent selective lung ventilation (SLV), and 60 patients (69.8%) underwent conventional non-selective ventilation. The mean age of the patients undergoing SLV was 14 years (5–17), and 30.7% were female. Patients in the SLV group had a significantly higher mean age (14.04 ± 3.35 years) compared to the non-selective ventilation group (10.68 ± 4.27 years, *p* < 0.05). This finding may indicate a preference for selective ventilation in older children, potentially due to their larger anatomical structures facilitating the technique. In the group of patients who underwent selective lung ventilation, there were three of 26 patients (11.5%) under 6 years of age. Three of the patients (11.5%) in this group with probable cyst rupture had less difficulty during the surgery, and one patient had a persistent air leak. However, none of the patients required reoperation.

The distribution of cysts in the right and left lung did not differ significantly between the groups (*p* = 0.138), suggesting that the choice of ventilation method was independent of cyst location.

There was a statistically significant difference in operative time between patients with and without selective ventilation (*p* < 0.001). The group with selective ventilation had significantly shorter operative times than the other group. Patients who underwent selective ventilation had a significantly shorter hospital stay than the other group (*p* < 0.001) (Table-1).

Importantly, there was no significant difference between the two groups in long-term air leaks or the need for reoperation (*p* > 0.05) (Table 1). Although the mean cyst volume was significantly larger in the selective ventilation group (221.6 ± 423.5 cm³) compared to the non-selective group (104.9 ± 106.2 cm³, *p* = 0.048), patients who underwent selective ventilation had significantly shorter operative times and fewer complications, including persistent air leaks. This finding highlights that selective ventilation may provide superior surgical exposure and more effective lung isolation, ultimately enhancing surgical efficiency and potentially reducing postoperative morbidity.

**Table 1:** Selective vs. Non-Selective lung ventilation: comparison of demographic and clinical outcome

	Selective Ventilation (*n* = 26)	Non-selective ventilation (*n* = 60)	*P*
**Gender**			
Female	8	23	0,502
Male	18	37	
**Age** (Mean ± Std Dev.)	14,04 ± 3,35	10,68 ± 4,27	**0**,**001**
**Side**			
Right Lung	12	38	0,138
Left Lung	14	22	
**Cyst Volume** (cm3) (Mean ± Std Dev.) (cm^3^)	221,6 ± 423,5	104,9 ± 106,2	**0**,**048**
**Operation time** (Mean ± Std Dev.) (min)	115,7 ± 37,7	153,7 ± 27,3	**0**,**000**
**Complication**; n			
Prolonged air leak (> 7 d)	1	4	0,608
Reoperation	0	3	0,549
**Length of stay hospital** (Mean ± Std Dev.) (days)	7,2 ± 2,6	12,3 ± 7,3	**0**,**001**

Regardless of cyst volume, selective ventilation reduced the length of hospital stay and the duration of surgery. Hospital length of stay was significantly shorter for patients in the selective ventilation group (7.2 ± 2.6 days) compared to those in the non-selective group (12.3 ± 7.3 days, *p* = 0.001). This further highlights the beneficial effects of selective ventilation in terms of faster postoperative recovery and reduced hospitalization time.

## Discussion

This study evaluates the outcomes of selective versus non-selective lung ventilation techniques in pediatric pulmonary hydatid cyst surgery. The side of lung involvement (right or left) did not significantly influence the choice of ventilation technique. The mean cyst volume was significantly larger in the selective ventilation group compared to the non-selective group (221.6 vs. 104.9 cm³), suggesting that selective ventilation is particularly advantageous in the management of larger cysts, likely due to the need for better exposure and isolation during surgery. The operative time was significantly shorter in the selective ventilation group compared to the non-selective group. Selective ventilation appears to enhance surgical efficiency by offering enhanced operating conditions and reducing intraoperative challenges without increasing postoperative complications, including prolonged air leak and reoperation. Furthermore, the mean length of hospital stay was significantly shorter in the selective ventilation group compared to the non-selective group. These findings support the potential benefits of selective ventilation in facilitating the surgical procedure and reducing hospital stay, which should be a reliable component of pulmonary hydatid cyst surgery.

The use of selective ventilation in the pediatric patient population presents some challenges due to anatomical and physiological differences [2]. Lung isolation techniques are limited by the smaller dimensions of the pediatric airway. Mainstream intubation is less preferred in older children and adolescents, especially if they can accommodate a double-lumen endobronchial tube (DLT) [16]. The smallest available DLT is 26 Fr, which may be suitable for children aged over 8–10 years [17]. For infants and young children, the main options are endobronchial intubation and the use of bronchial blockades. Selection of the appropriate size of endotracheal tube or bronchial blockade is essential for both initial and maintenance one-lung ventilation. Moreover, our study excluded patients whose airways were too small to accommodate available SLV equipment, significantly narrowing the generalizability of our findings. Additionally, the lack of appropriately sized endobronchial tubes and bronchial blockers for every pediatric age group further emphasizes the limitations in universally applying SLV techniques across all pediatric populations. Future studies and technological advancements should focus on addressing equipment size constraints to broaden the clinical applicability of selective lung ventilation.

In our study, patients in the selective ventilation group were significantly older (10.68 vs. 14.04 years) than those in the non-selective group, which may reflect a preference for selective ventilation in older children due to their larger anatomical structures that facilitate the technique. However, these inherent demographic and anatomical differences potentially confound direct outcome comparisons between the two groups. Although shorter operative times and hospital stays were observed in the SLV group despite these larger cyst volumes, caution should be exercised when interpreting these findings. Additionally, the absence of standardized patient-selection criteria for SLV introduces potential operator-dependent biases and limits the reproducibility of the results. Establishing clear, standardized selection guidelines or protocols, along with matched or stratified patient groups based on age and cyst volume, would mitigate these confounding factors in future studies, providing clearer and more robust evidence regarding the specific benefits of selective lung ventilation.

Numerous studies have demonstrated the benefits of selective lung ventilation in pediatric patients undergoing intrathoracic surgery, especially in younger patients [3, 18–20]. The application of selective lung ventilation has reached a significant milestone because of technological advancements. The use of Fogarty catheters and other methods for bronchial blockade has been replaced by more advanced endobronchial tubes and blockers that are specifically intended for pediatric use [21, 22]. In our study, three patients (11.5%) in the selective lung ventilation cohort were under the age of 6 years and were successfully ventilated selectively using Coopdech bronchial blocker. Our research suggests that selective lung ventilation techniques can be used effectively as an appropriate option for younger individuals. Nevertheless, the very small number of patients under the age of 6 years (*n* = 3) managed with bronchial blockers in our study limits the generalizability and strength of conclusions regarding the broad applicability of this technique in younger pediatric populations. Further dedicated studies with larger sample sizes focusing specifically on younger children are necessary to conclusively establish the safety and effectiveness of bronchial blockers in this subgroup.

The primary challenges related to selective lung ventilation include selecting the appropriate size of tube or bronchial blocker, ensuring and maintaining adequate isolation, and managing hypoxemia during the perioperative period with single-lung ventilation. Postoperative complications are often associated with atelectasis and pneumonia [3]. Our complication rates and operative times are consistent with those reported in the literature. No perioperative complications were observed, as intraoperative full lung expansion via ventilation allowed for the identification and resolution of potential issues such as residual air leaks. In our study, the operative length was significantly reduced in the SLV group, even though the cyst volume (221.6/104.9 cm3) was significantly larger in the SLV group than in the NSLV group. However, our study lacks detailed intraoperative data, including blood loss volumes, specific ventilation parameters, and surgeon-reported quality of operative exposure, due to its retrospective nature. These data would provide clearer insight into the exact mechanisms by which SLV improved operative efficiency and outcomes. Future prospective studies should systematically collect such intraoperative metrics to better understand and quantify the benefits associated with selective lung ventilation.

We believe that the combination of selective lung ventilation and controlled drainage of air from the obstructed lung led to the successful repair of bronchopulmonary fistulas and contributed to a reduction in operative time. Additionally, the ability to confirm the presence of other fistulas or assess the effectiveness of the repair when the blockage is removed thanks to controlled blockade is another significant benefit [23]. These findings suggest that selective ventilation techniques may be expanded in pediatric surgical practice and lead to improved patient outcomes.

Finally, the use of selective lung ventilation should be considered a valuable tool in high-risk and infectious pediatric thoracic procedures, particularly in children. While the study demonstrates the effectiveness of selective lung ventilation, several limitations should be noted. The retrospective nature of our study led to patient selection based on clinical judgment, age, and anatomical feasibility, rather than randomization, introducing a potential selection bias and limiting causal interpretations. Specifically, the assignment to SLV or NSLV groups was driven by patient age and technical feasibility, which further weakens the strength of causal conclusions drawn from our findings. Additionally, the relatively small sample size, especially in the selective ventilation group (*n* = 26), further limits the generalizability of our findings. Moreover, the limited number of patients in the SLV group restricts our statistical power to detect rare complications and prevents meaningful subgroup analyses. Future larger-scale, multicenter studies would be beneficial to adequately assess the safety profile of SLV, particularly regarding infrequent postoperative complications. Our analysis was limited to short-term perioperative outcomes, and we did not evaluate long-term respiratory function, cyst recurrence rates, or quality-of-life measures. Future longitudinal studies with extended follow-up periods are warranted to provide comprehensive insights into the long-term benefits and potential risks associated with selective lung ventilation in pediatric hydatid cyst surgery.

As a conclusion, to the best of our knowledge, this is the first study to evaluate the clinical effects of a selective lung ventilation technique in pediatric hydatid cyst surgery. Contamination of the contralateral lung with infected material and repair of air leaks are significant challenges in pulmonary hydatid cyst surgery. Our findings demonstrate that the selective ventilation technique significantly reduces operative time and hospital stay in patients who undergo the procedure, while maintaining a comparable complication rate.

These results were achieved because selective ventilation better isolates the surgical field and minimizes potential complications. Technological advances and the development of selective ventilation methods have enabled the safe implementation of these techniques in pediatric surgical practice. Our study demonstrated that selective ventilation effectively reduces operative time and hospital stay in thoracotomies for hydatid cysts of the lung and is a successful treatment option in children.

## Data Availability

No datasets were generated or analysed during the current study.
